# Supplementary material for: Exploring the Role of Desmoplastic Physical Stroma in Pancreatic Cancer Progression Using a Three-Dimensional Collagen Matrix Model
Source: Bioengineering (Basel). 2023 Dec 18;10(12):1437. doi: 10.3390/bioengineering10121437 (PMC10741102; doi:10.3390/bioengineering10121437)
Supplement: Supplementary file 1 [file bioengineering-10-01437-s001.zip › bioengineering-2764467-supplementary.pdf]

Table S1. Gene ontologies with the top 5 highest *p*-values of biological process in Collagen-Capan-1 model

GOID	Term	Count	p-value	Genes symbol
GO:0007165	signal transduction	37	0.008926	FBN2, ECM1, CXCL8, PDE1A, C5AR1, ADM, FGF1, NDRG1, RND1, AKAP12, IL1RL1, RASD1, RASSF10, INPP5D, TNFRSF8, SFN, RALGDS, IGFBP1, MAP2K3, FGFBP1, CCL22, TNFSF14, OSGIN1, GAB3, MCC, RHOD, GEM, GREM1, NR4A3, TAFA3, IL1B, GNB4, PKP1, HCLS1, RIN1, ASB2, TRIM55
GO:0045944	positive regulation of transcription from RNA polymerase II promoter	36	0.006002	CSRNP1, DLX2, FGF1, ETS1, RELB, SERTAD1, MYC, ZNF544, KDM6B, OSR2, MIR9-1HG, EGR2, EGR3, JAG1, EGR4, RFX2, NFATC2, BHLHA15, ARID3B, MAFA, BMP6, DCN, GREM1, FOSL1, BMP2, IL6, GAL, NR4A3, MAFB, IL1B, DDIT3, FOSB, HCLS1, ATF3, PPARD, ENG
GO:0008284	positive regulation of cell proliferation	28	2.76E-06	CSF2, PDGFB, PDGFA, ADM, CRIP2, FGF1, THBS1, AREG, PTHLH, SERTAD1, MYC, IL6R, OSR2, PDGFRA, EDN2, EGR4, BMP6, GREM1, FOSL1, BMP2, IL6, IL1B, CLCF1, SPDYA, HCLS1, CD248, ATF3, CRLF2
GO:0000122	negative regulation of transcription from RNA polymerase II promoter	27	0.04943	DLX2, NOTCH4, NRARP, BACH2, MYC, SFN, HES2, OSR2, PLK3, MSX2, NFATC2, DKK1, BMP6, BMP2, NR4A3, MAFB, DDIT3, ZNF439, SNAI1, CRY1, FOSB, HCLS1, SNAI2, MAFK, ATF3, PPARD, ENG
GO:0010628	positive regulation of gene expression	25	3.06E-05	CSF2, CXCL8, PDGFB, FGF1, ETS1, ACTG1, RELB, CDH5, MYC, LMNA, OSR2, NFATC2, DKK1, BMP6, ADAM19, SFRP4, BMP2, IL6, IL1B, TMEM119, PKP1, VIM, ATF3, PPARD, ENG

Table S2. Gene ontologies with the top 5 lowest *p*-values of biological process in Collagen-Capan-1 model

GOID	Term	Count	p-value	Genes symbol
GO:0006357	regulation of transcription from RNA polymerase II promoter	52	1.17E-06	ZNF892, MESP2, CSRNP3, RORC, HOXA13, MECOM, TRPS1, HOXA7, PITX2, HOXA6, SOX7, ZNF763, HOXA5, ZNF443, MEF2C, EBF2, ARID5B, PAX6, ETV2, ZNF75D, ISL1, FOXP2, RUNX2, TOX3, NR5A2, FOXD4L6, ZNF519, IRF2, HOXB3, HOXB8, ZFPM2, KDM7A, HOXB5, ZNF792, DLX1, TSHZ1, ZFP14, PRDM16, HOXC5, ZNF345, BCL11B, BICRAL, POU6F2, NR2F1, MEIS2, NFATC4, BCL6, NFIB, TLX2, ZNF737, JMY, SP5
GO:0045944	positive regulation of transcription from RNA polymerase II promoter	36	1.32E-04	DLX1, CSRNP3, GATA5, HOXA13, SOX2, MECOM, PRDM16, MYB, HOXA7, PITX2, ZNF345, HOXA5, NCOA2, MEF2C, BCL11B, NR2F1, EBF2, TET1, PAX6, ETV2, ISL1, MEIS2, RUNX2, RGMA, NFATC4, TOX3, NR5A2, NFIB, TLX2, IRF2, TLR9, HOXB3, RARB, MAML3, ZFPM2, HOXB5
GO:0000122	negative regulation of transcription from RNA polymerase II promoter	33	3.79E-05	DLX1, RORC, GATA5, SOX2, TRPS1, PRDM16, MYB, HOXA7, PITX2, ZNF345, NCOA2, SKOR1, MEF2C, SEMA4D, PTCH1, NR2F1, TET1, ARID5B, PAX6, ISL1, NR0B2, MEIS2, FOXP2, H1-3, NFATC4, BCL6, NFIB, IRF2, SP5, HOXB3, RARB, HOXB8, ZFPM2
GO:0007155	cell adhesion	23	3.47E-05	ENTPD1, PCDHGB6, VCAM1, SEMA4D, PCDH9, PCDHGA4, FAM24B-CUZD1, PCDH20, PCDHGC3, PCDHGA1, PCDHB11, PCDHGA9, SELP, ISLR, PCDHB6, CASS4, MXRA8, APBA1, CNTN3, SVEP1, PCDHB3, CD34, FREM2
GO:0006355	regulation of transcription, DNA-templated	23	0.047321	NCOA2, SKOR1, MEF2C, HCG_1984214, POU6F2, EBF2, PAX6, ZNF75D, MEIS2, RUNX2, SOX2, ZFP14, NR5A2, ZNF618, ZNF519, ZNF737, IRF2, MYB, RARB, PITX2, ZNF763, SOX7, ZNF443
